# Perceived Organizational Effectiveness during a Public Health Crisis and Moral Wellness among Nurse Leaders: A Cross-Sectional Study

**DOI:** 10.1155/2024/6808266

**Published:** 2024-05-09

**Authors:** Cynda Rushton, Katie Nelson, Alanna Bergman, Danielle Boyce, Christian Jenkins, Sandra M. Swoboda, Ginger Hanson

**Affiliations:** ^1^Johns Hopkins University School of Nursing, Baltimore, MD, USA; ^2^Johns Hopkins Berman Institute of Bioethics, Baltimore, MD, USA; ^3^Johns Hopkins Bloomberg School of Public Health, Center for Indigenous Health, Baltimore, MD, USA; ^4^Johns Hopkins University School of Medicine, Center for Data Science in Emergency Medicine, Baltimore, MD, USA; ^5^Johns Hopkins University School of Medicine, Baltimore, MD, USA

## Abstract

**Background:**

During a public health crisis, such as the COVID-19 pandemic, nurse leaders coordinate timely high-quality care, maintain profit margins, and ensure regulatory compliance while supporting the health and wellbeing of the nursing workforce. In a rapidly changing environment where resources may be scarce, nurse leaders are vulnerable to moral injury; however, organizational effectiveness may help to buffer moral challenges in healthcare leadership, thereby fostering greater moral resilience and reducing turnover intention.

**Aim:**

To understand mechanisms by which perceived organizational effectiveness contributes to nurse leaders' moral wellness (i.e., moral injury and moral resilience) and thereby effects work outcomes (i.e., engagement, burnout, and turnover intention).

**Methods:**

A cross-sectional survey of nurse leaders (*N* = 817) from across the United States was conducted using a snowball methodology, independent *t*-tests, and structural equation modeling to examine theoretical relationships among moral injury, moral resilience, and organizational effectiveness.

**Results:**

Higher ratings on every facet of perceived organizational effectiveness were significantly related to greater moral resilience (*p* < 0.001 for all *t*-tests) and lower moral injury (*p* < 0.001 for all *t*-tests) among nurse leaders. Structural equation models indicated both moral resilience and moral injury were significant mediators of the relationship between organizational effectiveness and work outcomes. Moral resilience and moral injury significantly mediated the effect of organizational effectiveness on burnout. Moral resilience was also a significant mediator of the relationship between organizational effectiveness and moral injury.

**Conclusion:**

Dismantling organizational patterns and processes in healthcare organizations that contribute to moral injury and lower moral resilience may be important levers for increasing engagement, decreasing burnout, and reducing turnover of nurse leaders.

## 1. Introduction

In public health crises, nurse leaders and frontline nurses are faced with health-related situations, which may overwhelm their capacities to address them [1]. The COVID-19 pandemic is the most recent example in the history of a global public health crisis, which severely strained existing public health infrastructure, taxed hospital resources, and degraded an already tenuous workforce [2–5]. During these situations, nurse leaders have a vital role in upholding organizations' missions and values, while concurrently leading staff through rapidly changing environments [6]. This requires not only deft skills in communication, leadership, compassion, and resolve but also an ability to prioritize wellbeing and resilience for themselves and their frontline staff. Nurse leaders must support the provision of high-quality care and represent frontline staff at the organizational level, all while serving as ethical role models [7]. During the COVID-19 pandemic, the challenges nurses faced at the point of care were highlighted [8]. However, the toll the pandemic took on nurse leaders was largely underreported. Many nurse leaders' work occurs “behind the scenes,” and as such, their voices and experiences are underreported in the literature with limited acknowledgment by their organizations ([9]—in press) and, therefore, are poorly understood.

### 1.1. Moral Injury

Nurse leaders must navigate a myriad of ethical and moral challenges and adversities that may cause moral suffering and contribute to burnout, which can lead to turnover intention and moral injury (MI) [10, 11]. These experiences may be particularly heightened amid a public health crisis [8, 9]. When moral suffering is conceived as a continuum, MI is viewed as the most corrosive type of moral suffering ([12], p. 52–76; 2023). MI occurs when persons perceive that they or others have violated their moral core producing symptoms, such as stress, burnout, and turnover intention [13]. Often, it includes betrayals or transgressions by self or others, particularly financial and administrative leaders [4], and can be associated with post-traumatic stress disorder, suicide ideation/attempts, and other mental health symptoms [2, 14]. Among nurses, studies have shown clinically significant MI symptoms (>36) among frontline nurses given their high-intensity work environment(s) and physical/emotional strain that comes with providing patient care [15, 16]; however, the degree of MI among nurse leaders in these situations is largely unexplored. Frontline nursing staff are not experiencing the burden of the pandemic alone [8]; the impact on nurse leaders is likely also significant. A 2022 survey by the American Organization for Nursing Leadership (AONL) found that emotional health of staff and retention are two of the three top challenges nurse leaders were facing in response to the pandemic. Of the AONL survey respondents, 38% of the nurse leaders are “maybe” or “definitely” leaving their roles, of which 43% identified the reason as burnout/exhaustion [17].

### 1.2. Moral Resilience

Moral resilience (MR) is “the capacity of an individual to restore their integrity in response to moral adversity” ([12], p. 68). Moral resilience encompasses personal integrity, relational integrity, buoyancy, self-regulation/awareness, moral efficacy, and self-stewardship [18]. Moral resilience has been posited as a protective resource that can reduce the detrimental effect of moral suffering [19]. A strengths-based approach, MR, has been shown to be inversely related to MI [16] and moral distress [5]. Higher levels of moral resilience are inversely correlated with stress, anxiety, and depression [5] and decreases in burnout and turnover intention [10]. Understanding the relationship of MR in response to potentially morally injurious events during a pandemic can illuminate opportunities for prevention and intervention.

### 1.3. Organizational Effectiveness

Nurse leaders are in a precarious position, balancing staff, and personal wellbeing while also meeting regulatory requirements and organizational expectations [20]. During a public health crisis like the COVID-19 pandemic, leaders depend upon the organization's structures, processes, and governance mechanisms to achieve desired outcomes. Organizational effectiveness (OE) is a complex measure of how well an organization maintains and delivers on its mission and services, supports personnel, and establishes a culture of employee commitment and satisfaction [21]. In contrast, less effective organizations are characterized by reduced professional autonomy and perceived value within the organization, and greater exposure to circumstances that violate and eventually erode integrity and moral capability [22]. During the pandemic, OE was constrained and contributed to symptoms of MI. A study of nurses, physicians, advanced practice providers, and others showed that facets of OE contributed to both MI and MR [16]. As organizational effectiveness was degraded, symptoms of MI increased, and MR decreased [16].

Foreshadowing the pandemic, the National Academy of Sciences, Engineering, and Medicine (NASEM) established goals for healthcare systems to assess the impact of system demands on employees and improve employee wellbeing [23]. A contributing factor to clinician wellbeing includes practicing in alignment with ones' professional values and working in an environment that enables ethical practice [24]. Cultivating an ethical climate that fosters wellbeing and MR is a challenge that requires a multipronged approach at both the individual and organizational levels [4, 25, 26]. Leading organizations, such as NASEM [23], the U.S. Surgeon General [27], the National Institute for Occupational Safety and Health [28], and the United Nations [3], have identified key priority areas, which include mental health support for healthcare providers and frameworks for systemic change, further echoing the call for action within healthcare institutions. The adoption of these recommendations in the United States and globally was in its infancy when the pandemic began in March 2020. Effective strategies for expanded occupational health and safety that support frontline staff and nurse leaders require further scientific inquiry [29]. Therefore, the purpose of this study was to examine the following four research questions among a sample of nurse leaders in the United States in the aftermath of the COVID-19 pandemic.

1. Are there differences in MR and MI based on whether nurse leaders rate different facets of OE as high or low during a public health crisis? Is nurse leaders' perceived OE associated with differences in MR and MI?
2. Does the effect of OE on work outcomes during a public health crisis (i.e., engagement, burnout, and turnover intention) operate through its negative effect on MI among nurse leaders?
3. Does the effect of OE during a public health crisis on work outcomes (i.e., engagement, burnout, and turnover intention) of nurse leaders operate through its positive effect on MR?
4. Does the effect of OE on MI operate through its positive effect on MR among nurse leaders during a public health crisis?

## 2. Methods

### 2.1. Study Design, Setting, and Participants

**Figure 1:**
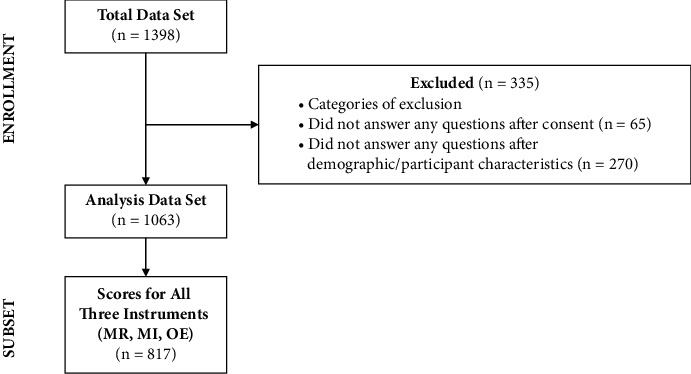
CONSORT diagram.

We conducted a cross-sectional survey via Qualtrics with a sample of nurse leaders from across the United States who were practicing during the COVID-19 pandemic (2020–2022). Using snowball sampling, we recruited nurse leaders in the United States, leveraging members of the American Organization of Nurse Leaders (AONL) to recruit its members, as well as the PI's professional networks (Figure 1). Participants were recruited via email and other communications sent by AONL and the research team, inviting them to complete the online survey. Participants were encouraged to share the survey with other nurse leaders within their networks. Data were collected from August through November of 2022. Inclusion criteria were as follows: (1) serving in a nurse leader role, as defined by the following categories: Chief Executive Officer, Chief Nursing Officer/Chief Nursing Executive, Chief Operating Officer, Vice President, Director, Manager, Specialist/Coordinator, Clinical Staff, Dean/Professor, Consultant, Vendor, or Other Nurse Leader; (2) practicing since at least 2019; (3) living in the United States; and (4) over the age of 18 years. Qualitative data obtained via open-ended questions from this survey were analyzed and published separately [9]. Elements of those data are described in the discussion to further contextualize and explain the quantitative findings. The Johns Hopkins Institutional Review Board deemed this study to be exempt; survey completion implied consent to participate.

### 2.2. Variables

#### 2.2.1. Demographics and Work Characteristics

All demographic and work characteristic covariates were categorical; therefore, each was dummy coded for inclusion in preliminary regression analyses and final path analyses. Age included five categories; education had three categories; and religion had five categories. For all analyses, role was recoded into the following three categories: nurse manager (reference group), executive, and other nurse leader. Race included five categories; primary work population included three categories; and time in current role had six categories.

#### 2.2.2. Organizational Effectiveness

OE was measured via 18 items adapted from a prior study involving HCWs during the pandemic [16]. The process for adapting the scale involved literature review, input from nurse leaders practicing during the pandemic, and responses from an AONL-sponsored focus group. The original items (*N* = 10) were included in addition to eight new items that were specific to nurse leaders during the COVID-19 pandemic. Participants rated items from 1 (not at all effective) to 5 (extremely effective). An exploratory factor analysis using principal axis factoring was conducted. A single factor was extracted, which explained 57.91% of the variability in the items. All communalities were greater than or equal to 0.50. All factor loadings were greater than or equal to 0.70. The exploratory factor analysis provided evidence for the construct validity of the scale. The total score was computed by taking the mean of all items, whereby higher scores indicate higher OE. The Cronbach's alpha reliability for this sample was 0.96.

#### 2.2.3. Moral Injury

The Moral Injury Symptoms Scale-Healthcare Professionals (MISS-HP) comprises 10 items rated on a scale from 1 (strongly agree) to 10 (strongly disagree) to assess MI symptoms [15]. The total score is computed by summing the items, with scores ranging from 10 to 100; higher scores indicate higher MI. The Cronbach's alpha reliability for our study was 0.71.

#### 2.2.4. Moral Resilience

The Rushton Moral Resilience Scale-16 (RMRS-16) is a 16-item scale rated on a 4-point Likert scale from 1 (disagree) to 4 (agree) to assess moral resilience [30]. The scale is divided into four subscales as follows: (1) response to moral adversity; (2) personal integrity; (3) relational integrity; and (4) moral efficacy—each of which includes four items. The total RMRS score was computed by averaging item responses, where a higher score indicates greater moral resilience with scores ranging from 1 to 4. Overall reliability for the total RMRS in our study was 0.85.

#### 2.2.5. Work Engagement

We used a modified 8-item version of the Utrecht Work Engagement Scale-9 (UWES-9) [31]. Participants rated frequency on a scale from 1 (never) to 6 (always, everyday). Total score was computed by averaging the items, where higher scores indicate greater work engagement, and scores range from 1 to 6. Reliability for our study was 0.91.

#### 2.2.6. Burnout

We used a 4-item scale validated by Profit and colleagues [32] to measure emotional exhaustion as a component of burnout. Items were rated on a scale from 1 (strongly disagree) to 5 (strongly agree). The total score was configured by averaging the four items, subtracting 1, and multiplying by 25 such that scores range from 0 to 100 where higher scores indicate higher burnout. Cronbach's alpha for our study was 0.90.

#### 2.2.7. Turnover Intention

Turnover intention was measured using the 3-item “leave job” subscale developed by Dotson and colleagues [33]. Wording was changed from “nursing” to “nursing leadership” to make items more relevant to the population. Items were rated on a scale from 1 (strongly disagree) to 5 (strongly agree). The score was computed by averaging the three items; higher scores indicate higher turnover intention. Cronbach's alpha for our study was 0.93.

### 2.3. Statistical Methods

Data were analyzed using STATA Release 18 (StataCorps; College Station, TX). Frequency analyses were run to describe the overall sample. We began by recoding the OE facets so that low OE = not at all/slightly/moderately effective and high OE = very/extremely effective to assess relationships to moral resilience and MI. We then used independent *t*-tests with each of the 10 facets of OE as independent variables and moral resilience and MI as dependent variables. We reported the Cohen's D effect size for each *t*-test.

Structural equation models (SEMs) were generated to estimate complex models with multiple mediation effects, while controlling for other hypothesized relationships to circumvent any potential bias. We tested three mediation relationships: 1) organization effectiveness > moral resilience > work outcome; (2) OE > MI > work outcome; and (3) MR > MI > work outcome. We ran separate models for each work outcome (burnout, work engagement, and turnover intention) because the theoretical relationships between these variables were not the primary focus of this analysis. Goodness-of-fit indices used to evaluate the overall model were the chi-square test, RMSEA, p-close, CFI, and TFI. Guidelines for what represents a good fit included were as follows: (1) a chi-square value that is not statistically significant; (2) RMSEA values of ≤0.06 [34, 35]; (3) P-close values of greater than 0.05 since this is a test of whether RMSEA is significantly greater than 0.05; and (4) CFI and TFI values of ≥0.95 [34, 35].

We used the *medsem* command in Stata, a postestimation command run after estimating each model, to generate estimates of each indirect (mediation) effect and associated standard error and used bootstrapping [36]. The *medsem* command also generates effect sizes for the indirect effect referred to as RIT and RID [37]. The RIT effect size is a ratio of the indirect effect to the total effect and is interpreted as the percentage of the effect of the independent variables on the dependent variable (direct effect) that goes through the mediator. RID is a ratio of the indirect effect and the direct effect, and it is interpreted as the proportion larger the indirect or mediation effect is as opposed to the direct effect.

Before running the SEM models, we ran preliminary simple linear regression analyses to determine which demographic and work characteristics to use as covariates for each outcome (i.e., MR, MI, work engagement, burnout, and turnover intention) in the path analyses. Any of these variables that were significantly related to an outcome at this stage were included as a predictor of that variable in the path analysis.

## 3. Results

### 3.1. Participants

The study included a total sample of 1063 nurse leaders from across the United States, of which 817 individuals completed online surveys for MR, MI, and OE, and this was our final analysis sample for this study. Of the 817 individuals who completed all three surveys, 90.1% were females and 89.3% were White. Most leaders (84.8%) were between 36 and 65 years of age and had graduate degrees (86.6%). The race and ethnicity of this nursing leader sample was largely White (89.3%) and non-Hispanic (95.6%). Over half (58.9%) worked with adult patients, and 31.6% reported being in their current role for less than three years (Table 1). The three main roles held by the participants were as follows: Chief/VP (24.8%), Director (32.2%), and Manager (29.4%).

### 3.2. Relationship of OE with MR and MI

A series of independent *t*-tests with Cohen's D effect sizes were conducted to examine whether there were differences in MR and MI between nurse leaders who rate different facets of OE as high or low (Table 2). For every facet of OE, nurse leaders who rated their organization as high on that facet experienced higher moral resilience (*p* < 0.001 for all *t*-tests) and lower MI (*p* < 0.001 for all *t*-tests) than nurse leaders who rated their organization as low on that facet. All effect sizes were in the medium to large range (absolute values = |0.47|–|0.91|), indicating that all are likely important contributors to nurse leaders' wellbeing. One facet,an environment that promotes speaking up about concerns without fear of retaliation, stood out with relatively high effect sizes for both moral resilience (Cohen's *D* = 0.68) and MI (Cohen's *D* = −0.91). The facet, “protocols for filling staffing needs when current staff have fulfilled their assignments”, was most strongly related to nurse leader's MR (Cohen's D = 0.72). Facets that were most strongly associated with MI (absolute values of Cohen's *D* < |0.70|) included the following: (a) forums with leaders to whom I report to share concerns (Cohen's *D* = −0.76); (b) pathways for requesting ethics consultation or advice (Cohen's *D* = −0.77); and (c) an environment that promotes speaking up about concerns without fear of retaliation (Cohen's *D* = −0.91).

### 3.3. Path Analysis of the Relationships of OE, MR, and MI with Work Outcomes

#### 3.3.1. Identifying Covariates

Preliminary simple linear regression models were run to determine whether any of the demographic or work characteristics were associated with one of the outcomes of the path analysis models. Potential covariates examined included the following: age, gender, LGBTQ identity, education, religion, occupational role, race, ethnicity, work population, work setting, and length of time in current role. Outcomes for these simple linear regressions included the following: MR, MI, work engagement, burnout, and turnover intention. Covariates that were significantly related to MR included the following: age, education, role, race, work population, and tenure in role. Those covariates significantly related to MI were age, education, religion, and role. Covariates that were significantly related to work engagement included the following: age, education, role, and work population. The following covariates were significantly related to burnout: age, education, and role. The covariates that were significantly related to turnover intention were age and role. We included covariates that were related to an outcome as covariates for that outcome in the path analyses (A, B, and C).

#### 3.3.2. A: Path Analysis for Work Engagement

**Figure 2:**
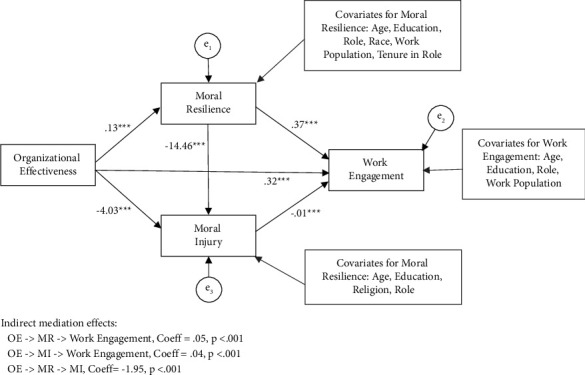
Path analysis of the relationship among OE, MR, MI, and work engagement of nurse leaders (*N* = 800).

SEM was used to simultaneously test our three competing mediation hypotheses for work engagement (i.e., (1) OE- > MR- > work engagement; (2) OE- > MI- > work engagement; and (3) MR- > MI- > work engagement) (Figure 2). The covariates included for each outcome variable in the model are listed in Figure 2. All goodness-of-fit indices indicated a good fit for the overall model (Table 3). All main direct paths in the model were statistically significant (Table 4).

The following is a summary of the hypothesized mediation effects for the path analysis for work engagement.

We examined whether moral resilience mediated the relationship between OE and work engagement controlling for the other predictors in the model. The indirect or mediating effect was statistically significant, coeff = 0.05, se = 0.01, and *p* < 0.001. The RIT effect size indicates that 14% of the effect of OE on engagement is mediated by MR.

We examined whether MI mediated the relationship between OE and work engagement controlling for the other predictors in the model. The indirect or mediating effect was statistically significant, coeff = 0.04, se = 0.01, and *p* < 0.001. The RIT effect size indicates that about 12% of the effect of OE on engagement is mediated by MI.

We examined whether MR mediated the relationship between MI and work engagement controlling for the other predictors in the model. The indirect or mediating effect was statistically significant, coeff = −1.95, se = 0.23, and *p* < 0.001. According to the RIT effect size, 33% of the effect of OE on MI is mediated by MR.

#### 3.3.3. B: Path Analysis for Burnout

**Figure 3:**
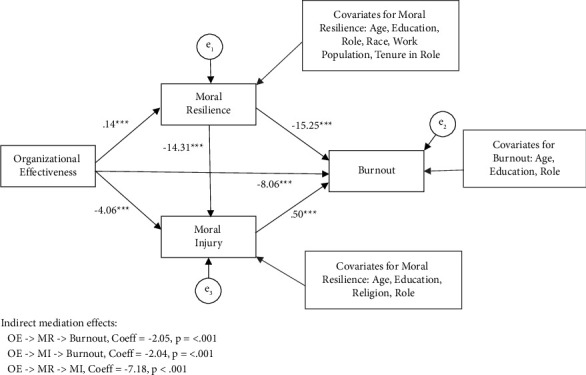
Path analysis of the relationship among OE, MR, MI, and burnout of nurse leaders (*N* = 800).

Next, SEM was used to simultaneously test our three competing mediation hypotheses for burnout: (1) OE- > MR- > burnout; (2) OE- > MI- > burnout; and (3) MR - > MI - > burnout. The covariates included for each outcome variable in the model are listed in Figure 3. All goodness-of-fit indices indicate a good fit for the overall model (Table 3). All main direct paths in the model were statistically significant (Table 5). The following is a summary of the hypothesized mediation effects.

We examined whether MR mediated the relationship between OE and burnout controlling for the other predictors in the model. The indirect or mediating effect was statistically significant, coeff = −2.05, se = 0.40, and *p* < 0.001. The RIT effect size indicates that 20% of the effect of OE on engagement is mediated by moral resilience.

We examined whether MI mediated the relationship between OE and burnout controlling for the other predictors in the model. The indirect or mediating effect was statistically significant, coeff = −2.04, se = 0.40, and *p* < 0.001. The RIT effect size indicates that about 20% of the effect of OE on engagement is mediated by MI.

We examined whether MR mediated the relationship between MI and burnout controlling for the other predictors in the model. The indirect or mediating effect was statistically significant, coeff = −7.18, se = 1.32, and *p* < 0.001. According to the RIT effect size, 32% of the effect of OE on MI is mediated by MR.

#### 3.3.4. C: Path Analysis for Turnover Intention

**Figure 4:**
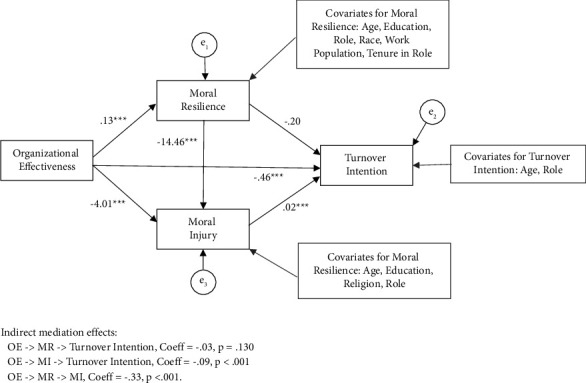
Path analysis of the relationship among OE, MR, MI, and turnover intention of nurse leaders (*N* = 799).

Finally, SEM was used to simultaneously test our three competing mediation hypotheses for turnover intention: 1) OE- > MR- > turnover intention; (2) OE- > MI- > turnover intention; and (3) MR- > MI- > turnover intention. The covariates included for each outcome variable in the model are listed in Figure 4. All goodness-of-fit indices indicate a good fit for the overall model (Table 3). All main direct paths in the model, except for moral resilience to turnover intention, were statistically significant (Table 6). The following is a summary of the hypothesized mediation effects.

We examined whether MR mediated the relationship between OE and turnover intention controlling for the other predictors in the model. The indirect or mediating effect was not statistically significant, coeff = −0.03, se = 0.02, and *p*=0.130.

We examined whether MI mediated the relationship between OE and turnover intention controlling for the other predictors in the model. The indirect or mediating effect was statistically significant, coeff = −0.09, se = 0.02, and *p* < 0.001. The RIT effect size indicates that about 20% of the effect of OE on engagement is mediated by MI.

We examined whether MR mediated the relationship between MI and turnover intention controlling for the other predictors in the model. The indirect or mediating effect was statistically significant, coeff = −0.33, se = 0.07, and *p* < 0.001. According to the RIT effect size, 62% of the effect of OE on MI is mediated by MR.

## 4. Discussion

The goal of this study was to explore the relationship between OE and work outcomes, such as engagement, burnout, and turnover intention among U.S. nurse leaders, considering MI and MR in hypothesized pathways. We found that all facets of OE significantly contributed to MR and MI, as demonstrated by medium/large effect sizes, indicating a strong link between OE and nurse leaders' moral wellness (MI and MR). These findings parallel those of a previous study with frontline nurses, which found 9 of the 10 facets of OE significantly contributed to both MI and MR [16]. Nurse leaders, particularly during a crisis, must prioritize staff wellbeing while also meeting regulatory mandates, financial objectives, and other organizational priorities to remain competitive in their professional environments. The inability to advocate for staff needs due to organizational change erodes leaders' integrity and their ability to navigate workplace challenges [38]. This is consistent with other studies that suggest that OE is predictive of MI symptoms and can be modified to reduce the detrimental impact [16, 19]. As noted in our previously published qualitative work, nurse leaders used open-ended survey questions as an opportunity to describe OE and its impact on ethical decision-making, which are referenced below to provide supporting context for key findings.

The facet of OE most strongly correlated with nurse leaders' MR was having protocols for addressing staffing needs when current staff have fulfilled their assignments. During the COVID-19 pandemic, shortages of nurses and other healthcare workers created difficult ethical challenges related to the allocation of scarce human resources. When filling staffing gaps, a common practice is to ask those already working to work overtime, which creates tension between leaders and staff who feel overworked. In the open-ended questions, leaders described this dilemma, *“middle-level management was drug through the mud during the pandemic. We received the pressures from both above and from our staff, and nothing was ever enough*” (Clinical Staff Leader). When healthcare organizations have clear protocols to avoid this conflict, MR is amplified; when protocols are absent, MR can be eroded, particularly when resources are constrained by a public health crisis. Another leader expanded on the lack of guidance and support in making ethical decisions, *“there is little support to make one feel confident in making decisions and the fear of getting in trouble is always looming over my head. Little guidance given, never any clear guidelines but always judged after the fact”* (Nurse Manager). These sentiments align with organizations like AONL, who are advocating for greater investment in nursing administration research to strengthen the provision of clear protocols and guidance for nurse leaders to utilize within their institutions [39]. Likewise, using an ethical framework for human resources allocation can make explicit the ethical tradeoffs that are necessary and illuminate the rationale for them [40].

Nurse leaders felt excluded from organizational decisions regarding staffing during the COVID-19 pandemic, which contributed to feelings of disempowerment and distress. Open-ended comments, such as “directions come from above that are polar opposite of what I have voiced, and my teams are feeling. This adds to additional challenges and stress” (Clinical Director), illustrated this concern. Executive leaders responsible for OE and outcomes may have adopted a “command and control” response rather than a collaborative, inclusive process for decision-making that equitably included nurse leaders. When nurse leaders are excluded from decision-making or their expertise and feedback are seemingly disregarded, trust is inherently broken [4, 41]. From this research, we posit that when nurse leaders are involved with staffing and nursing care delivery decisions (especially during a pandemic), and when there are clear and transparent protocols for addressing surges in care, their moral efficacy, response to moral adversity, relational integrity, and self-stewardship are amplified. When nurse leaders are properly resourced and engaged in decision-making, their ethical commitments and wellness are maximized and trust is fostered. As a matter of justice and fairness, asking people who are already depleted to fill staffing gaps creates threats to relational integrity with their team and their ability to live their core values, such as respect for persons, fairness, and equity. Having protocols and processes that honor employee boundaries and commitments without coercion or retribution, especially during a public health crisis, can potentially relieve the moral suffering of both nurse leaders and frontline nurses [13].

Trust has consistently been identified as a key factor in mitigating the negative impact(s) of ineffective work environments on nurses. A qualitative analysis by Nelson et al. [4] yielded evidence, which supports the need for organizational infrastructure and supports from leaders to rebuild broken trust in the workplace. They offered the following five key remedies for rebuilding trust: (1) counseling/emotional support; (2) peer-to-peer support; (3) education and ethical support; (4) wellness offerings; and (5) spiritual/faith support. Given the nature of MI, rebuilding organizational trust may be an important element in restoring integrity and sustaining the nursing workforce, especially in the aftermath of a pandemic [42]. Models such as the Reina 3 Cs for trust building offer leaders a roadmap for identifying where trust is being built and broken and specific behaviors that are needed to sustain or rebuild it when it is broken [41].

The facets of OE that were most strongly associated with MI included the following: a) a lack of forums with other leaders to share concerns; (b) an environment that prohibits voicing concerns for fear of retaliation; and (c) the absence of pathways for requesting ethics consultation or advice. Taken together, these items suggest that nurse leaders' MI is decreased when there are strong and safe paths of communication within the leadership structure where they can share ethical concerns and obtain support for ethical decision-making from peers and experts. When nurse leaders lack psychologically safe space to share concerns, trust can erode, and MI symptoms may ensue. Although nurse leaders are outspoken advocates for patients and staff, their expertise is undervalued and nursing voices are often silenced, “*they (organizational leaders) don't want to hear criticism and you can't fix problems if all you hear is silence. Nurse leaders should not just hold a seat at the table, they should feel free to express ideas and concerns*” (Clinical Educator). In contrast, leaders who had a voice and contributed to decision-making felt supported and prepared for the ethical challenges brought by the pandemic. “*I truly believe that our organization has all of the disciplines at the table when making decisions and we were prepared to continue to lead through a crisis with strong integrity and professionalism*” (Clinical Director). These findings suggest that innovative, trustworthy structures, and processes are required to create the conditions for nurse leaders to be heard, understood, and their expertise valued.

Lack of access to ethics consultation or advice has the potential to intensify MI symptoms, especially when nurse leaders are grappling with complex and uncertain ethical questions for which there are no easy answers [43]. During the COVID-19 pandemic, ethical issues were no longer episodic but rather imbedded in everyday reality. Lacking skills or processes to systematically address these issues via dialogue with others likely resulted in the accumulation of moral residue [44]. Over time, unresolved ethical issues can contribute to feelings of moral ineffectiveness, moral suffering including symptoms of MI, and compromised integrity. Whitehead et al. [45] found that moral distress was negatively correlated with ethical workplace climate among a sample of 592 clinicians. The highest sources of moral distress were watching patient care suffer due to lack of continuity and poor communication among care team members [45]. Strengthening access to ethics consultants, creating proactive mechanisms to identify ethical concerns, and fortifying ethics education for nurse leaders may help mitigate these types of negative consequences. Doing so may also strengthen the relational integrity of the entire team [24, 44].

Both MR and MI were mechanisms through which OE was associated with work engagement and burnout. Higher levels of OE were associated with greater MR and less MI, which was in turn related to better work engagement and less burnout. Previous research has demonstrated that OE is inversely related to MI [24]. The inverse relationship between MR and burnout has also been confirmed in other kinds of research [10]. In addition, an intervention to increase elements of MR was related to increases in work engagement [30]. This research builds upon the previous research outlined by examining a more integrated model of how these constructs are related to one another. This suggests there are several intervention points that are likely to amplify the impact on outcomes, such as work engagement and burnout.

When considering both MR and MI as mechanisms through which OE impacts turnover intention, MI was a more important mediator of OE on turnover intention. Previous research has found an inverse relationship between MR and turnover intention [10]; however, constructs were not previously examined in a larger statistical model with OE. One nurse manager described an organizational culture of blame and its impact on their mental and moral wellness and ultimately their decision to leave. “*The organization I left was unforgiving. The (leadership) was very into blame, ‘fix it' and unsupportive. I was rung out. I had nothing left. It was horrible to leave the wonderful team I had worked so closely with through the pandemic, but I had to leave that hospital... or mentally implode*” (Nurse Manager). These types of cases may cause leaders to seek a position elsewhere, regardless of their MR, where they may be better supported to make decisions in alignment with their values. The exodus of nurse leaders postpandemic has led to a concerning trend that further threatens the sustainability of the U.S. nursing workforce [42].

In addition, when organizations were more effective, nurse leaders reported greater MR, and this mechanism was associated with lower MI. Thus, some of the effects of MR on work outcomes are indirect through its relationship with MI. Specifically, greater OE is associated with greater MR and lower MI, which is thereby related to higher work engagement, less burnout, and lower turnover intention. This is consistent with other findings that demonstrate the role of MR as a protective resource to decrease the detrimental impact of OE in key areas [10]. This does not imply amplifying workers' tolerance for unethical situations but to restoring their moral efficacy to choose what is in alignment with their values and commitment to acting in integrity-preserving ways. As such, in cases where perceived OE is low, MR tends to be lower and may contribute to higher turnover intention. Our previous work also found that greater MR was associated with lower MI [24]; however, this study expands on those findings by how MR may mediate the relationship between OE and MI.

### 4.1. Limitations

This study did have limitations that should be considered when considering interpretation and generalization of the results. This was a convenience sample; thus, respondents may have been those who were particularly interested in OE and wellbeing during the pandemic. That said, recruiting via AONL, a national organization, and encouraging participants to share the initiation with others did result in representation from all 50 U.S. states. This, in addition to the large sample size, makes it likely that the sample is reasonably representative of nurse leaders in the United States. Whether these findings are relevant in other countries is beyond the scope of this study but warrants further investigation. The study was cross-sectional in nature; therefore, we could not establish the time-order relationships of mediational relationships. For that reason, we have done our best to ground the proposed paths in prior research and theory. In addition, all data were self-reported and, thus, relationships may be inflated due to common method variance.

## 5. Conclusion

Nurse leaders are experiencing MI symptoms related to OE. Healthcare organizations must focus on dismantling institutional structures and processes that negatively impact the moral wellbeing of leaders. MR and MI are both important pathways through which OE impacts workplace engagement and burnout. In other words, OE is related to increased MR and decreased MI, which may influence greater work engagement and less burnout among nurse leaders. However, to achieve these more favorable outcomes, nurse leaders' moral suffering must be given sustained attention. Promoting a work environment that enables voicing concerns without fear of retaliation is a critical starting point for fostering respect and trust needed to ultimately retain nurse leaders in their positions. Future research should strive to understand the dynamic interplay of investments needed to create healthier work environments for both nurse leaders and frontline nurses collectively [46, 47].

## Figures and Tables

**Table 1:** Demographic characteristics of nurse leaders (*N* = 817).

Variable	*N*	%
Age (*N* = 817)		
18–35 years old	46	5.6
36–45 years old	167	20.4
46–55 years old	244	29.9
56–65 years old	282	34.5
Over 65 years old	78	9.5
Gender (*N* = 812)		
Male	74	9.1
Female	738	90.9
LGBTQ (*N* = 806)		
No	749	92.9
Yes	57	7.1
Education (*N* = 817)		
Bachelor's or less	126	15.4
Masters	445	54.5
Doctorate	246	30.1
Religion (*N* = 817)		
No religious preference	93	11.4
Christian/Protestant	365	44.7
Roman Catholic	230	28.2
Spiritual but not religious	86	10.5
Other	43	5.3
Role (*N* = 817)		
Chief/VP	203	24.8
Director	263	32.2
Manager	240	29.4
Other	35	4.3
House supervisor	11	1.3
Clinical leader	65	8.0
Race (*N* = 806)		
White	720	89.3
Black	33	4.1
Asian	23	2.9
Other	18	2.2
Multiple races	12	1.5
Hispanic (*N* = 813)		
Yes	36	4.4
No	777	95.6
Primary work population (*N* = 816)		
Pediatric	68	8.3
Adult	481	58.9
Both	267	32.7
Primary work setting (*N* = 809)		
Hospital, short-term acute care	371	45.9
Hospital, long-term acute care	14	1.7
Post-acute care facility (IRF, SNF, CCRC)	10	1.2
Specialty hospital	11	1.4
Health system facility	112	13.8
Health system corporate office	39	4.8
Academic healthcare setting	151	18.7
Critical access hospital	23	2.8
Behavioral health facility	8	1.0
Outpatient, community-based clinic	19	2.3
Ambulatory surgery, specialty care facility	8	1.0
Free-standing emergency, urgent care facility	6	0.7
Other health care setting	37	4.6
Primary work setting location (*N* = 813)		
Urban	440	54.1
Suburban	259	31.9
Rural	114	14.0
Primary practice location (*N* = 581)		
Emergency department	50	8.6
Inpatient—critical care	118	20.3
Inpatient—other	287	49.4
Operating room	24	4.1
Outpatient/ambulatory care	102	17.6
Length of time in current role (*N* = 817)		
Less than 3 years	258	31.6
About 3–5 years	211	25.8
About 5–10 years	171	20.9
About 10–15 years	81	9.9
About 15–20 years	51	6.2
Greater than 20 years	45	5.5

**Table 2:** Association of low vs. high OE with MR and MI.

Item	OE	Moral resilience				Moral injury			
		*N*	*M* (SD)	*p*	Cohen's *D*	*N*	*M* (SD)	*p*	Cohen's *D*
Information regarding professional wellness resources	Low	412	3.17 (0.41)	<0.001	0.47	412	36.87 (13.09)	<0.001	−0.70
	High	404	3.36 (0.40)			404	28.25 (11.66)		

Policies regarding crisis response (e.g., the role of triage officers/triage teams)	Low	439	3.17 (0.42)	<0.001	0.52	439	36.41 (13.33)	<0.001	−0.68
	High	364	3.38 (0.39)			364	27.99 (11.36)		

Forums with leaders to whom I report to share concerns	Low	407	3.14 (0.41)	<0.001	0.64	407	37.15 (13.00)	<0.001	−0.76
	High	400	3.40 (0.39)			400	27.88 (11.42)		

Information regarding hazard supplemental compensation	Low	499	3.18 (0.41)	<0.001	0.58	499	35.75 (13.23)	<0.001	−0.67
	High	235	3.41 (0.41)			235	27.32 (11.36)		

Opportunities for individual or team-based approach to address stress	Low	484	3.18 (0.41)	<0.001	0.50	484	36.00 (13.07)	<0.001	−0.68
	High	328	3.39 (0.41)			328	27.54 (11.55)		

Pathways for requesting ethics consultation or advice	Low	408	3.14 (0.41)	<0.001	0.64	408	37.27 (12.92)	<0.001	−0.77
	High	397	3.40 (0.39)			397	27.85 (11.41)		

Information regarding confidential reporting mechanisms	Low	309	3.14 (0.41)	<0.001	0.50	309	38.09 (13.02)	<0.001	−0.72
	High	489	3.35 (0.41)			489	29.20 (12.04)		

An environment that promotes speaking up about concerns without fear of retaliation	Low	333	3.11 (0.40)	<0.001	0.68	333	39.01 (13.20)	<0.001	−0.91
	High	465	3.38 (0.40)			465	28.09 (11.00)		

Communication updates regarding system-based changes	Low	333	3.13 (0.40)	<0.001	0.55	333	37.59 (13.18)	<0.001	−0.68
	High	464	3.36 (0.41)			464	29.07 (11.97)		

Psychological and emotional support for leaders	Low	564	3.19 (0.41)	<0.001	0.64	564	35.27 (13.12)	<0.001	−0.69
	High	247	3.45 (0.39)			247	26.60 (11.09)		

Policies for increasing the number of ICU beds	Low	408	3.17 (0.42)	<0.001	0.54	408	36.05 (13.29)	<0.001	−0.64
	High	320	3.38 (0.38)			320	28.05 (11.48)		

Policies or processes for redeployment of staff	Low	453	3.16 (0.41)	<0.001	0.58	453	35.96 (13.36)	<0.001	−0.63
	High	336	3.40 (0.40)			336	28.07 (11.32)		

Processes for staff to “call-out” without retribution	Low	422	3.17 (0.42)	<0.001	0.51	422	35.99 (13.32)	<0.001	−0.59
	High	371	3.38 (0.40)			371	28.59 (11.80)		

Proactive training of staff to be “crosstrained” to work in multiple areas	Low	460	3.17 (0.42)	<0.001	0.58	460	35.69 (12.91)	<0.001	−0.61
	High	338	3.40 (0.39)			338	28.11 (11.98)		

Protocols for filling staffing needs when current staff have fulfilled their assignments	Low	533	3.17 (0.41)	<0.001	0.72	533	35.44 (12.81)	<0.001	−0.69
	High	273	3.46 (0.37)			273	26.85 (11.57)		

Transparent communication regarding policy or practice changes	Low	409	3.16 (0.40)	<0.001	0.52	409	36.75 (12.87)	<0.001	−0.69
	High	401	3.38 (0.41)			401	28.29 (11.77)		

Budget adjustments to increase resources for nursing workforce	Low	473	3.17 (0.41)	<0.001	0.57	473	35.84 (13.03)	<0.001	−0.63
	High	330	3.40 (0.40)			330	27.95 (11.92)		

Equitable compensation for nurses in same role	Low	487	3.17 (0.41)	<0.001	0.58	487	35.73 (13.20)	<0.001	−0.64
	High	311	3.41 (0.39)			311	27.63 (11.53)		

*Note.* Cohen's *d* effect sizes: |0.2| = small; |0.5| = medium; |0.8| = large.

**Table 3:** Goodness-of-fit indices for path analysis models for the relationships of OE, MR, MI, and work outcomes.

Fit indices	Model for work engagement (*N* = 800)	Model for burnout (*N* = 800)	Model for turnover intention (*N* = 799)
Chi-square (df), *p* value	37.04 (28), 0.118	39.10 (30), 0.123	36.30 (32), 0.275
RMSEA	0.02	0.02	0.01
*p*-close	1.000	1.000	1.000
CFI	0.99	0.99	0.99
TLI	0.98	0.98	0.99

**Table 4:** Coefficients, standard errors, and *p* values for path analysis models for the relationships of OE, MR, MI, and work engagement (*N* = 800).

	Coefficient	SE	*p*		

Moral resilience					
Organizational effectiveness	0.13	0.01	<0.001		
Moral injury					
Moral resilience	−14.46	0.96	<0.001		
Organizational effectiveness	−4.03	0.39	<0.001		
Work engagement					
Moral resilience	0.37	0.08	<0.001		
Moral injury	−0.01	0.003	<0.001		
Organizational effectiveness	0.32	0.03	<0.001		

Mediation effects	Indirect effect coefficient (Monte Carlo)	SE	*p*	Effect sizes for indirect effects	
				RIT	RID

OE -> MR -> work engagement	0.05	0.01	<0.001	0.14	0.16
OE -> MI -> work engagement	0.04	0.01	<0.001	0.12	0.13
OE -> MR -> MI	−1.95	0.23	<0.001	0.33	0.48

**Table 5:** Coefficients, standard errors, and *p* values for path analysis models for the relationships of OE, MR, MI, and burnout (*N* = 800).

	Coefficient	SE	*p*		

Moral resilience					
Organizational effectiveness	0.14	0.01	<0.001		
Moral injury					
Moral resilience	−14.31	0.96	<0.001		
Organizational effectiveness	−4.06	0.39	<0.001		
Burnout					
Moral resilience	−15.25	2.65	<0.001		
Moral injury	0.50	0.09	<0.001		
Organizational effectiveness	−8.06	1.01	<0.001		

Mediation effects	Indirect effect coefficient (Monte Carlo)	SE	*p*	Effect sizes for indirect effects	
				RIT	RID

OE -> MR -> burnout	−2.05	0.40	<0.001	0.20	0.26
OE -> MI -> burnout	−2.04	0.40	<0.001	0.20	0.25
OE -> MR -> MI	−7.18	1.32	<0.001	0.32	0.47

**Table 6:** Coefficients, standard errors, and *p* values for path analysis models for the relationships of OE, MR, MI, and turnover intention (*N* = 799).

	Coefficient	SE	*p*		

Moral resilience					
Organizational effectiveness	0.14	0.01	<0.001		
Moral injury					
Moral resilience	−14.46	0.96	<0.001		
Organizational effectiveness	−4.01	0.39	<0.001		
Turnover intention					
Moral resilience	−0.20	0.13	0.121		
Moral injury	0.02	0.004	<0.001		
Organizational effectiveness	−0.46	0.05	<0.001		

Mediation effects	Indirect effect coefficient (Monte Carlo)	SE	*p*	Effect sizes for indirect effects	
				RIT	RID

OE -> MR -> turnover intention	−0.03	0.02	0.130	0.06	0.06
OE -> MI -> turnover intention	−0.09	0.02	<0.001	0.17	0.20
OE -> MR -> MI	−0.33	0.07	<0.001	0.62	1.66

## Data Availability

The data used to support the findings of this study are available from the corresponding author upon reasonable request.
